# Investigating neurological symptoms of infectious diseases like COVID-19 leading to a deeper understanding of neurodegenerative disorders such as Parkinson's disease

**DOI:** 10.3389/fneur.2022.968193

**Published:** 2022-12-07

**Authors:** Jing Zhang

**Affiliations:** Department of Neurology, School of Medicine, Washington University in St. Louis, St. Louis, MO, United States

**Keywords:** COVID-19, Parkinson's disease, neurological symptoms, neurodegenerative disorders, post-COVID-19 parkinsonism management and prevention

## Abstract

Apart from common respiratory symptoms, neurological symptoms are prevalent among patients with COVID-19. Research has shown that infection with SARS-CoV-2 accelerated alpha-synuclein aggregation, induced Lewy-body-like pathology, caused dopaminergic neuron senescence, and worsened symptoms in patients with Parkinson's disease (PD). In addition, SARS-CoV-2 infection can induce neuroinflammation and facilitate subsequent neurodegeneration in long COVID, and increase individual vulnerability to PD or parkinsonism. These findings suggest that a post-COVID-19 parkinsonism might follow the COVID-19 pandemic. In order to prevent a possible post-COVID-19 parkinsonism, this paper reviewed neurological symptoms and related findings of COVID-19 and related infectious diseases (influenza and prion disease) and neurodegenerative disorders (Alzheimer's disease, PD and amyotrophic lateral sclerosis), and discussed potential mechanisms underlying the neurological symptoms and the relationship between the infectious diseases and the neurodegenerative disorders, as well as the therapeutic and preventive implications in the neurodegenerative disorders. Infections with a relay of microbes (SARS-CoV-2, influenza A viruses, gut bacteria, etc.) and prion-like alpha-synuclein proteins over time may synergize to induce PD. Therefore, a systematic approach that targets these pathogens and the pathogen-induced neuroinflammation and neurodegeneration may provide cures for neurodegenerative disorders. Further, antiviral/antimicrobial drugs, vaccines, immunotherapies and new therapies (e.g., stem cell therapy) need to work together to treat, manage or prevent these disorders. As medical science and technology advances, it is anticipated that better vaccines for SARS-CoV-2 variants, new antiviral/antimicrobial drugs, effective immunotherapies (alpha-synuclein antibodies, vaccines for PD or parkinsonism, etc.), as well as new therapies will be developed and made available in the near future, which will help prevent a possible post-COVID-19 parkinsonism in the 21st century.

## Introduction

It has been reported during the coronavirus disease 2019 (COVID-19) pandemic that around a third of patients with COVID-19 suffered from neuropsychiatric disorders (1), and 82% of patients hospitalized with COVID-19 had neurological symptoms such as headache, loss of sense of smell and/or taste, encephalopathy, cognitive impairment, impaired consciousness and stroke (2). COVID-19 is caused by infection with severe acute respiratory syndrome coronavirus 2 (SARS-CoV-2). Viral infections such as infection with herpes simplex virus 1 (HSV-1), influenza A virus or human immunodeficiency virus (HIV) can trigger neuroinflammation (which initiates neurodegeneration) (3), and increase the risk of Alzheimer's disease (AD) (4), Parkinson's disease (PD) (5), or amyotrophic lateral sclerosis (ALS) (6). SARS-CoV-2 infection has been found to induce neuroinflammation (7), and increase the risk of AD and PD in susceptible individuals (8–11). One of the consequences of SARS-CoV-2 infection is lasting neuroinflammation and subsequent neurodegeneration in long COVID (11–13).

Historically, there was an outbreak of encephalitis lethargica and a global spreading of parkinsonism following the 1918 influenza pandemic, and people who were born during the 1918 influenza pandemic had a 2~3 times higher risk of developing PD than those born before 1888 or after 1924. Reexamination of the 1918 influenza pandemic data revealed that the influenza A virus was involved in PD (14). In addition, a recent study indicated that influenza was related to Parkinson's disease over 10 years following infection (odds ratio: 1.73), i.e., influenza infection was likely to increase the long-term risk of Parkinson's disease (15). These findings have highlighted the need to investigate COVID-19 neurological symptoms, complications and their mechanisms in the COVID-19 pandemic.

A number of cases have been reported that patients developed acute parkinsonism (several days or weeks) after contracting SARS-CoV-2 (16–22), and a large cohort study indicated that COVID-19 survivors had an estimated incidence of 0.11% for parkinsonism in the following 6 months after their COVID diagnosis (1). In addition, recent research found that SARS-CoV-2 nucleocapsid protein accelerated alpha-synuclein aggregation (*in vitro*) (23), and the presence of SARS-CoV-2 existed in the substantia nigra of 6 COVID-19 patients (24). Further, SARS-CoV-2 infection caused dopaminergic neuron (derived from human pluripotent stem cells) senescence (both *in vitro* and *in vivo*) (24), worsened preexisting PD symptoms in around 59% of PD patients (25), and increased susceptibility to parkinsonism induced by oxidative stress (9). These findings suggested that SARS-CoV-2 infection increases individual vulnerability of PD or parkinsonism, leading to the onset or progression of PD (or parkinsonism) or in some cases unmasking preexisting pre-symptomatic PD in susceptible individuals (26).

Searching for possible links between COVID-19 and PD or parkinsonism (9, 26, 27), clinicians and medical researchers have been asking, discussing and debating: Will there be a post-COVID-19 parkinsonism after the COVID-19 pandemic (11, 26, 28–31)? There are currently 10 reported COVID-19 associated acute parkinsonism cases (16–22), but according to the estimated incidence of 0.11% for parkinsonism among COVID-19 survivors in the large-cohort (*n* = 236,379) long follow-up study (1), 260 of them developed parkinsonism 6 months after COVID onset (1), which provided strong evidence and support for post-COVID-19 parkinsonism. The estimated incidence of 0.20% for parkinsonism among the COVID-19 survivors who were hospitalized due to COVID-19 together with the 0.11% incidence for parkinsonism among all the COVID-19 survivors in the large-cohort (1) further suggested that there would be much more post-COVID-19 parkinsonism cases than COVID-19 associated acute parkinsonism cases globally. Furthermore, this study revealed that there was no significant difference between the probability of developing post-infection parkinsonism in COVID-19 survivors after SARS-CoV-2 infection and that in influenza survivors after influenza infection (1).

There was emerging evidence of a Parkinson's “pandemic” before the COVID-19 pandemic (e.g., the total number of PD cases increased 118% globally from 1990 to 2015 and doubled from around 3 million in 1990 to over 6 million in 2015) and it was estimated that the global burden of Parkinson's disease (accounting for changes in aging, industrialization, etc.) would be over 17 million by 2040 (32). The widespread SARS-CoV-2 infection during the COVID-19 pandemic increased the risk of PD or parkinsonism (9, 24, 26), either triggered parkinsonism (16, 19) or unmasked preexisting prodromal (or pre-symptomatic) PD in COVID-19 patients or survivors [especially in long COVID (1)] around the globe. In addition, since (1) a number of COVID-19 symptoms (loss of sense of smell, taste impairment, dizziness, etc.) and long COVID symptoms (cognitive impairment, tremors, functional mobility impairment, gastrointestinal problems, sleep disorder, etc.) (13, 33–35) overlap with symptoms of PD or parkinsonism, (2) the evidence of the COVID-associated acute parkinsonism cases (16–22), the 260 (0.11%) post-COVID-19 parkinsonism cases (1), high percentages of functional mobility impairment (44%) and mobility decline (20.2%) in long COVID (13), and an increasing trend of motor symptoms like tremors (35) among patients with long COVID has suggested that SARS-CoV-2 infection may trigger (acute or chronic) neural injury in the nigrostriatal system, a long lasting neuroinflammation and subsequent motor-associated neurodegeneration, (3) there are much similarities between SARS-CoV-2 and H1N1 influenza virus [in viral contagiousness/transmission, viral infection fatality/severity, immune system activation, cytokine storm induction, neuroinvasion, cellular aging pathway modulation, association with increased risk/susceptibility to PD or parkinsonism (9, 15), etc.], and the risk factors for the recent Parkinson's “pandemic” (such as aging and industrial/agricultural/chemical/metal pollution) still exist (32), there might be an increased global risk of PD or parkinsonism associated with long COVID and a possible post-COVID-19 parkinsonism (1). Therefore, the further question is: How to prevent a possible post-COVID-19 parkinsonism?

In order to raise public awareness of the neurological damage that SARS-CoV-2 infection induces and to prevent possible post-COVID-19 parkinsonism, this paper reviewed the neurological symptoms and related findings of COVID-19 and 2 other infectious diseases (influenza and prion disease) that are related to PD, parkinsonism or neurodegenerative movement disorder. In addition, since SARS-CoV-2 infection increases the risk of AD and PD neurodegeneration (8–12, 24), neurodegenerative disorders such as AD, PD and ALS were covered in this paper, which like COVID-19, also cause severe patient sufferings, numerous life loss, high healthcare cost and heavy social burden. Further, this paper discussed the potential mechanisms underlying the neurological symptoms of these diseases and the relationship between the infectious diseases and the neurodegenerative disorders as well as their therapeutic and preventive implications in the currently incurable neurodegenerative disorders.

## Neurological symptoms and related findings of the infectious diseases and the neurodegenerative disorders

The main neurological symptoms of the infectious diseases (COVID-19, influenza, and prion disease) and the neurodegenerative disorders (AD, PD, and ALS) and possible causes of these diseases are summarized in Table 1. For COVID-19 and PD, their main neurological symptoms and related findings are highlighted in this section.

**Table 1:** Main features (including neurological symptoms) of infectious diseases (COVID-19, influenza, and prion disease), and neurodegenerative disorders (Alzheimer's Disease, Parkinson's Disease, and Amyotrophic Lateral Sclerosis).

	**COVID-19**	**Influenza**	**Prion disease**	**AD**	**PD**	**ALS**
Incubation/prodrome period	1–14 days	1–5 days	~15 months to over 30 years	Years-decades	Years-decades	Months-years
Main types/subtypes	• COVID-19 variants: • Alpha, Beta, Gamma, Eta, …, • Delta, • Omicron	• Influenza A (causes the majority of influenza infection and pandemics/epidemics), • Influenza B, • Influenza C, • Influenza D	• Kuru; • CJD: • [sCJD (85%), fCJD, • and aCJD including vCJD, iCJD, etc.)]; • FFI; • FSE; • GSS			• Sporadic ALS (90–95%); Familial ALS • Classic ALS (70%); • PLS (5%); • PMA (5%); Regional variants
Main cells infected/affected in human	Epithelial cells in the respiratory tract	Epithelial cells in the respiratory tract	Lymphocytes, neurons, microglia, astrocytes	Neurons (in the hippocampus, entorhinal cortex, etc.), microglia, astrocytes	Dopaminergic neurons (in the substantia nigra), microglia, astrocytes	• Motor neurons, microglia, astrocytes
Neurological symptoms	(1) COVID-19: Smell and/or taste impairment (18.3–88.8%), weakness (27.9–53.5%), malaise (24.7–52.9%), fatigue (31.6–44.4%), encephalopathy (9.4–34.1%), myalgia (19.2–25.1%), sleep disorder (14.9–23.5%), headache (9.2–20.7%) and dizziness (11.3%) were common; cerebrovascular diseases (stroke, microvascular injury) (9.9%), GBS (6.9%), movement disorders (5.2%), impaired consciousness (3.8–9.6%), nausea (4.6–9.8%), seizures (0.06–4.05%), etc. were less common (35–40)	(1) Common: Encephalopathy (14.3–34.4%) (MERS, AESD, PRES, etc.), • Seizures (8–27.4%) (41); (2) Uncommon: • Aseptic meningitis; • Stroke (0.1–2.4%) (HSES); Myopathy; ANE; • Kleine-Levin syndrome; GBS (5.2%); • ADEM (0.1-−7%) (41); • Viral meningoencephalitis; Encephalitis lethargica; • Post-encephalitic Parkinsonism	• sCJD: ementia, Muscle stiffness; Difficulty speaking; • Movement disorders (Difficulty walking, • myoclonus, chorea, tremor); • Seizures; • Impaired vision, etc. • vCJD: Painful • sensory symptoms (dysesthesia), psychiatric symptoms (depression, anxiety, etc.), dementia, • several motor symptoms (poor coordination, chorea, etc.)	• Progressive memory decline (especially in short-term memory), cognitive impairment (or impaired executive function), language problem, disorientation, mood swings, etc.	(1) Motor: Tremors, bradykinesia, rigidity, difficulty walking, unstable posture; (2) Non-motor: • cognitive impairment, loss of smell and/or taste, gastrointestinal problems (such as nausea and constipation), dysautonomia, mood swings, sleep disorder and pain	• Muscle problems (weakness, stiffness, twitches or spasms, atrophy), pain (due to nerve damage), difficulty in walking, moving limbs, speaking, swallowing, breathing (at late stage)
	• Long COVID: • after 5 months, 54% had >1 symptom: functional mobility impairment (44%), fatigue or weakness (37.5%), pain (32.4%), sleep disorder (27%), cognitive impairments (17.1–23.8%), loss of smell and/or taste (11–13%), headache (8%) (13)					
Cause	SARS-CoV-2 infection	Influenza virus infection	• Unknow for sCJD (90%); • Genetic inheritance for fCJD (10%); Prion infection for vCJD, kuru, etc.; • Hypotheses such as • prion protein only hypothesis	• Mostly unknown; • Less than 5% due to genetic inheritance; Hypotheses: cholinergic hypothesis, amyloid hypothesis, tau hypothesis, • inflammatory hypothesis, infectious hypothesis; • Environmental factors: Psychosocial stress, etc.	• 5–10% due to gene mutation; • Environmental factors: Pesticide exposure; Head injury history; Drug-induced; Toxin-induced (e.g., manganese); • Vascular diseases (e.g., stroke) and neurodegenerative disorders (e.g., MSA) induced; • Infectious hypothesis	• Unknown; • Genetic factors (5-10% familial ALS); • Environmental factors: • Toxins (pesticides such as DDT); Exposure to lead and other metals; Alcohol and tabaco use; • Head injury history; • Sports (soccer, American football)
Pathogen infection causes cytokine storm?	Yes	Yes	No			
Neuroinflammation?	Yes (in some patients)	Yes (in some patients)	Yes	Yes	Yes	Yes
Neuroimmune impairment?	Yes (e.g., trigger GBS and MS relapses in some patients)	Yes (in some patients)	Yes	Yes	Yes (in most patients)	Yes
Neurodegeneration?	Yes (in some patients)	Yes (in some patients)	Yes	Yes	Yes	Yes

aCJD, acquired Creutzfeldt-Jakob disease; AD, Alzheimer's disease; ADEM, acute disseminated encephalomyelitis; AESD, acute encephalopathy with seizures and late restricted diffusion; ALS, Amyotrophic lateral sclerosis; ANE, acute necrotizing encephalitis; Anosmia, loss of smell or taste; CJD, Creutzfeldt-Jakob disease; CMV, Cytomegalovirus; CN, Cryptococcus neoformans; CNS, central nervous system; EBV, Epstein-Barr virus; fCJD, familial Creutzfeldt-Jakob disease; FFI, fatal familial insomnia; FSE, familial spongy encephalopathy; GBS, Guillain–Barré syndromes; GSS, Gerstmann-Sträussler-Scheinker syndrome; HSES, hemorrhagic shock and encephalopathy syndrome; HV, Herpes virus; iCJD, iatrogenic Creutzfeldt-Jakob disease; JCV, John Cunningham virus; MERS, mild encephalopathy with reversible splenial lesion; MSA, multiple system atrophy; PD, Parkinson's disease; PLS, primary lateral sclerosis; PMA, progressive muscular atrophy; PML, Progressive multifocal leukoencephalopathy; PRES, posterior reversible encephalopathy syndrome; sCJD, sporadic Creutzfeldt-Jakob disease; TGP, toxoplasma gondii parasite; TP, Treponema pallidum; vCJD, variant Creutzfeldt-Jakob disease.

### COVID-19

Meta-analyses showed that smell impairment (18.3–85.6%), taste impairment (19.6–88.8%), weakness (27.9–53.5%), malaise (24.7–52.9%), fatigue (31.6–44.4%), encephalopathy (9.4–34.1%), myalgia (19.2–25.1%), sleep disorder (14.9–23.5%), headache (9.2–20.7%), and dizziness (11.3%) were common among COVID-19 patients, while cerebrovascular diseases (ischemic or hemorrhagic stroke and microvascular injury) (9.9%), Guillain–Barré syndromes (GBS) (6.9%), movement disorders (5.2%), impaired consciousness (3.8–9.6%), nausea (4.6–9.8%), and seizures (0.06–4.05%) were less common (36–40). The prevalence of neurological symptoms is much higher in hospitalized patients with COVID-19: acute encephalopathy (49%), coma (17%), etc. (2). The estimated incidences in COVID-19 patients at the intensive care unit (ICU) were 9.58% for stroke, 4.24% for nerve (or nerve root) disorders, 3.35% for myoneural (or muscle) disease, 1.74% for dementia, and 0.26% for parkinsonism (1).

In addition, around 31–69% of patients with COVID-19 suffer from long COVID (13), and common post-acute COVID-19 symptoms in long COVID include fatigue, loss of concentration or memory, headache, dizziness, anosmia and gastrointestinal distress (42). A meta-analysis found that after 5 months, 54% of COVID-19 survivors experienced at least one post-acute symptom such as functional mobility impairment, fatigue or muscle weakness, pain, sleep disorder and cognitive impairment (Table 1) (13). Another study further indicated that after 6 months, a number of symptoms remained: fatigue, malaise, cognitive dysfunction, sensorimotor symptoms (including tremors and internal vibrations), headaches, memory problems, muscle (or joint) pain, sleep problems, and gastrointestinal disorders (including nausea and constipation) (34). Moreover, ~30% of patients had persistent symptoms 9 months after the onset of COVID-19 (43).

Further, imaging, lab, and pathological findings confirmed the neurological damage in the nervous system caused by SARS-CoV-2 infection. Imaging studies showed common abnormalities such as acute ischemic stroke lesions, white matter lesions, and hyperintensity and/or microhemorrhages in the olfactory bulb on magnetic resonant imaging (MRI) (44). A meta-analysis revealed that olfactory bulb abnormalities (23.1%) were the most common neuroimaging abnormality in COVID-19 patients, followed by white matter abnormality (17.6%), acute/subacute ischemic infarction (16%), and encephalopathy (13%) (45), while microhemorrhages, acute intracranial hemorrhage, and encephalitis were less common (46). Particularly, microvascular injury was observed in the olfactory bulb, brain stem and basal ganglia (including substantia nigra) in COVID-19 patients (47). In addition, the presence of SARS-CoV-2 was detected in the CSF of 6% patients who had acute neurological symptoms (48), in the brains (frontal lobe, basal ganglia, brain stem, cerebellum, etc.) of 21 (53%) deceased patients (49), and in the substantia nigra of 6 patients (24). Apart from the presence of SARS-CoV-2, post-mortem studies reported inflammation (35.6%), gliosis with diffuse activation of microglia and astrocytes (35.6%), arteriosclerosis (29.5%), hypoxic-ischemic injury (28.1%), diffuse edema (17.1%), intracranial bleed (subarachnoid hemorrhage and punctate hemorrhages) (12.4%) and infarctions (2.7%) in the brains of COVID-19 patients (7) (Table 2).

**Table 2:** Imaging, lab and pathology findings of neurological injury in the infectious diseases (COVID-19, influenza and prion disease) and the neurodegenerative disorders (Alzheimer's Disease, Parkinson's Disease, and Amyotrophic Lateral Sclerosis).

	**COVID-19**	**Influenza**	**Prion disease**	**AD**	**PD**	**ALS**
Imaging findings	May be normal; Common abnormalities: olfactory bulb abnormalities (23.1%), followed by white matter abnormality (17.6%), acute/subacute ischemic infarction (16%) and encephalopathy (13%) (45); Less common: microhemorrhages, acute intracranial hemorrhage, and encephalitis (46); Other lesions: optic nerve subarachnoid spaces sign, ADEM-compatible lesions, etc. (50)	May be normal; MERS: mid-splenial diffusion restricting lesion on DWI; AESD: T2 hyperintensity and diffusion restricting lesions, frontoparietal predominance; HSES: T2 hypointensity foci (suggesting hemosiderin deposition) mainly in cortex, basal ganglia, white matter (51); ANE: T2 hyperintensity and symmetric diffusion restriction (on DWI) in thalami, putamina, pariventricular white matter, midbrain, cerebellum (52); PRES: hyperintense signal on T2 MRI in semiovale, diffuse brain oedema. Post infectious cerebellitis: T2WI hyperintensity in the cerebellum, with brainstem compression and hydrocephalus; Encephalitis lethargica: atrophy in the midbrain, subthalamus and hypothalamus (53); Post viral parkinsonism: depigmentation of the substantia nigra and locus coeruleus	sCJD: Hyperintensity in the basal ganglia and/or cerebral cortex (the cortical ribbon-like hyperintensity) on DWI or T2/FLAIR MRI (24–42% showed only cortical lesions on DWI; 46–68% showed lesions in both the cortex and basal ganglia; 5–13% lesions only in the basal ganglia) (54). In addition, cerebellar atrophy in some patients; (2) vCJD: Over 75% showed the pulvinar and hockey stick sign—bilateral hyperintensity in the posterior thalamus on T2 MRI. Other areas may include dorsomedial thalamic nuclei and the periaqueductal gray matter (55).	MRI: Brain atrophy in the medial temporal lobe (hippocampus, entorhinal cortex and parahippocampal gyrus) and enlarged ventricles; DTI: White matter decline (diffusion anisotropy reduction); fMRI: Altered functional network connectivity and hippocampal activity; PET: Abnormal beta-amyloid and tau aggregation (56)	MRI: Brain atrophy in the basal ganglia and frontal lobe (57); Cortical thinning in brain regions such as orbitofrontal and ventrolateral prefrontal regions; SPECT/PET: Reduced dopamine transporter binding in the striatal or basal ganglia region (57, 58); Neuroinflammation in the basal ganglia; SWI: Loss of dorsolateral nigral hyperintensity (the “swallow tail” sign) (58); DTI: Reduced fractional anisotropy in the substantia nigra; fMRI: Altered functional network connectivity (57, 58)	MRI: Bilateral brain atrophy in the motor cortex and corticospinal tract, and enlarged ventricles, etc. (59); Hyperintensity in the cortical spinal tract on T2 MRI; fMRI: Cerebral activity alterations and altered functional connectivity in the sensorimotor network and cerebellum (60); PET: Abnormal cerebral glucose metabolism; Activated microglia in the frontal and motor regions; Reduced flumazenil- serotonin binding in the frontotemporal regions (61)
Lab findings	CSF: Normal, or (2%) with pleocytosis (2%), protein increase; the presence of SARS-CoV-2 in CSF in 6% patients who had acute neurological symptoms, and the presence of SARS-CoV-2 antibodies in 12% patients (48). Blood: lymphopenia, leukopenia, elevated CRP, increased LDH, elevated liver transaminases, elevated aspartate aminotransferase, increased levels of cytokines such as IL-6 (62)	CSF: 68% abnormal: increased white blood cell count (pleocytosis), elevated proteins; Influenza virus RNA in 16% of patients (63) Blood: may be normal, 71% abnormal: lymphocytopenia, leukocytosis, thrombocytopenia, elevated aspartate aminotransferase, elevated CRP (63)	CSF: May be normal; elevated S100B proteins in 84% sCJD patients; CSF 14-3-3 protein positive for 86% sCJD patients, but only in half of vCJD patients (64). The presence of prion PrPSc detected by RT-QuIC with ~89% accuracy (65). EEG: generalized periodic sharp wave pattern in 50% sCJD (66)	CSF: Decreased beta-amyloid (beta-amyloid peptide 1-42), increased tau (total tau, phosphorylated tau), etc. Blood: Beta-amyloid, plasma tau, Increased axonal protein neurofilament light, etc. (66) Urine: Possibly some free radicals, exogenous metabolites, cholesterol derived metabolites (lipid peroxidation compounds) (67)	CSF: Increased alpha-synuclein, etc. (68) Blood: Alpha-synuclein species and neurofilament light chain protein were detected (69)	CSF: Increased TDP-43 (70) EMG: fibrillation and positive sharp waves (denervation), and multispike and fasciculation potentials (neurogenic atrophy); Nerve conduction study: Abnormal motor nerve conduction (71)
Pathology findings	(1) The presence of SARS-CoV-2 in the brains (in regions such as frontal lobe, basal ganglia, brain stem and cerebellum) of 21 (53%) deceased patients (49) (2) Common: diffuse edema (17.1%), gliosis with diffuse activation of microglia and astrocytes (35.6%),	H1N1-AE: Leakage of plasma proteins from blood vessels to the brain, activated microglia, clasmato-dendrosis of astrocytes; Gross brain edema, high plasma IL-6 and IL-10 concentrations in some patients (72)	Prions found in the brain, spinal cord, and blood (1) In all subtypes: spongiform lesion (in gray matter), neuronal loss, deposition of abnormal misfolded prion, astrogliosis and microgliosis; (2) In certain subtypes: Amyloid plaques: abundant “florid plaques,” consisting of	Misfolded beta amyloid protein aggregation forming extracellular amyloid plagues in the hippocampus and its associated structure such as entorhinal cortex; Tau protein aggregation forming intracellular neurofibrillary tangles; neuronal loss; synapse loss (73)	Misfolded alpha-synuclein protein aggregation formed Lewy bodies; Nigral neurodegeneration combined with alpha-synuclein positive Lewy bodies and Lewy neurites in the substantia nigra, tau aggregation,	Misfolded TDP-43 protein aggregation in motor neurons causes loss of motor neurons in the spinal ventral horns, most brainstem motor nuclei and motor cortex; motor cortex atrophy; and spinal cord sclerosis (74)
	hypoxic-ischemic injury (28.1%) intracranial bleed (subarachnoid hemorrhage and punctate hemorrhages) (12.4%), infarctions (2.7%), arteriosclerosis (29.5%), inflammation (35.6%) (7) (3) Microvascular injury in the olfactory bulb, substantia nigra and brain stem (47).		amyloid plaques surrounded by vacuoles in vCJD and kuru; Phospho-tau deposition: in GSS and vCJD; Alpha-synuclein aggregation: in sCJD, iCJD and vCJD (75)		vascular lesions, and neuronal loss in the basal ganglia (76)	

AD, Alzheimer's disease; ADEM, acute disseminated encephalomyelitis; AESD, acute encephalopathy with seizures and late restricted diffusion; ALS, Amyotrophic Lateral Sclerosis; ANE, acute necrotising encephalitis; AST, aspartate aminotransferase; CNS, central nervous system; CRP, C-reactive protein; CSF, cerebrospinal fluid; DTI, diffusion tensor imaging; DWI, diffusion weighted imaging; EEG, Electroencephalography; EMG, Electromyogrphy; FLAIR, fluid-attenuated inversion recovery; fMRI, functional magnetic resonant imaging; GBS, Guillain–Barré syndromes; GSS, Gerstmann-Sträussler-Scheinker syndrome; HSES, hemorrhagic shock and encephalopathy syndrome; H1N1-associated encephalopathy, H1N1-AE; iCJD, iatrogenic CJD; IL, interleukin; MERS, mild encephalopathy with reversible splenial lesion; LDH, lactate dehydrogenase; MRA, magnetic resonant angiography; MRI, magnetic resonant imaging; MRS, magnetic resonant spectroscopy; NAA, N-acetylaspartate; PD, Parkinson's disease; PET, positron emission tomography; PMCA, misfolding cyclic amplification; PML, Progressive multifocal leukoencephalopathy; PRES, posterior reversible encephalopathy syndrome; RT-QuIC, Real-Time Quaking-Induced Conversion; sCJD, sporadic Creutzfeldt-Jakob disease; SWI, susceptibility weighted imaging; TDP-43, transactive response DNA binding protein 43 kDa; vCJD, variant Creutzfeldt-Jakob disease.

### Parkinson's disease

The symptoms of PD are 2-fold: (1) Motor symptoms such as tremors, bradykinesia, rigidity, difficulty walking, and unstable posture; (2) Non-motor symptoms such as cognitive impairment, loss of smell and/or taste, gastrointestinal problems (such as nausea and constipation), dysautonomia, mood swings, rapid eye movement (REM) sleep behavior disorder and pain. Some non-motor symptoms such as hyposmia, constipation and REM sleep behavior disorder often precede motor-symptoms. In addition, cognitive problems (such as dementia and language problems), depression and apathy become common at the late stage of the disease (Table 1).

Imaging showed a loss of hyperintensity in the bilateral substantia nigra, and atrophy in the basal ganglia and frontal lobe on MRI in advanced PD (58), a loss of dorsolateral substantia nigral hyperintensity (the “swallow tail” sign) on susceptibility-weighted imaging (SWI), and reduced dopamine transporter binding in the basal ganglia on dopamine transporter scan imaging SPECT (single-photon emission computed tomography) or PET (position emission tomography) (57, 58). Increased alpha-synuclein was detected in the CSF of patients with PD (68), and alpha-synuclein species and neurofilament light chain protein were detected in the blood of PD patients (69). Autopsy of deceased PD patients revealed neurodegeneration combined with alpha-synuclein positive Lewy bodies and Lewy neurites in the substantia nigra, tau aggregation, vascular lesions, and neuronal loss in the basal ganglia (76) (Table 2).

### COVID-19-associated acute and chronic PD (or parkinsonism)

The case reports that patients developed COVID-19-associated acute parkinsonism after contracting SARS-CoV-2 are summarized in Table 3. Clinical evidence suggested that SARS-CoV-2 infection was primarily responsible for such sudden parkinsonism (especially in young or middle-aged patients who had no prodromal PD symptoms) 16–22], but could not rule out the possibility that SARS-CoV-2 infection unmasked pre-symptomatic PD (21). Table 3 shows that (1) COVID-19-associated acute parkinsonism occurred in young, middle-aged and old patients 3–50 days after the onset of their mild, moderate or severe SARS-CoV-2 infection; (2) ~89% (8 of 9) patients did not have prodromal PD symptoms; (3) Among those whose dopaminergic imaging was available, 100% (5 of 5) patients had reduced dopaminergic uptake in the striatum; (4) ~63% (5 of 8) patients responded to dopaminergic medications and had improved motor symptoms after taking levodopa or dopamine agonist; (5) ~22 % (2 of 9) patients' motor symptoms improved spontaneously after either non-treatment (16) or convalescent plasma treatment (19) for SARS-CoV-2 infection. The possibility of SARS-CoV-2 infection unmasking prodromal (or presymptomatic) PD may exist in old patients who developed COVID-19 related acute parkinsonism (or PD) and had prodromal symptoms [e.g., constipation in the Baylor case (21)], but less likely in young or middle-aged patients who had no prodromal symptoms, and unlikely in the 2 cases where patients' motor symptoms improved spontaneously without any dopaminergic medication treatment (16, 19). Further research is needed to clarify the link between SARS-CoV-2 infection and PD.

**Table 3:** Summary of 10 COVID-19-associated acute parkinsonism cases (reported as of April, 2022).

**Case study**	**Pt gender**	**Pt age**	**COVID-19 severity**	**Days to parkinsonism after COVID onset**	**Parkinsonism symptoms**	**Imaging (dopaminergic uptake)**	**Prodromal symptoms**	**Response to dopamine promotor (e.g., levodopa)**
Mendez-Guerrero et al. (16)	M	58	Severe (ICU care)	32 days	• Rest and postural tremor, right hypokinetic-rigid syndrome	Reduced bilateral putamen uptake (especially in the left putamen)	None	No response to apomorphine (levodopa inhalation powder); Spontaneous improvement
Faber et al. (17)	F	35	Mild	10 days	Right rigidity, bradykinesia and hypophonia, hypomimia, slow saccades, gait impairment	Reduced left putamen uptake	None	Improvement after treatment with levodopa/benserazide
Cohen et al. (18)	M	45	Moderate (hospitalized)	2–3 weeks	Tremor (right > left), bradykinesia, rigidity	Reduced uptake in bilateral putamen (especially in the left putamen) and left caudate	None	Improvement after treatment with a dopamine agonist and anticholinergics
Akilli et al. (19)	M	72	Severe (ICU care)	3 days	Impaired walking, rest tremors, rigidity, bradykinesia	Not reported	Not reported	Not reported; Convalescent plasma was used to treat COVID-19; Motor symptom improved spontaneously
Rao et al. (20)	M	72	Severe (hospitalized)	14 days	Cog-wheel rigidity, postural instability, bradykinesia and falls	Not reported	None	Improvement after treatment with levodopa
	M	66	Moderate (hospitalized)	2 weeks	Cog-wheel rigidity, postural instability, bradykinesia and falls	Not reported	None	Mobility and speech improved after treatment with levodopa-carbidopa
	M	74	Severe (hospitalized)	3 weeks	Rigidity, postural instability and motor slowing	Not reported	None	Mobility, speech and swallowing improved after treatment with levodopa-carbidopa
Makhou et al. (21)	F	64	Mild	5 days	Rest tremor with hypomimia, Left bradykinesia, rigidity	Reduced right putamen uptake	Constipation	Unknown
Morassi et al. (22)	F	70	Moderate to severe (hospitalized)	6–7 weeks	Rigidity, bradykinesia, hypomimia, hypophonia, ophthalmoparesis	Reduced uptake in bilateral putamen (more severe in left putamen)	None	Modest response to levodopa/carbidopa, motor function improvement over time (walked with aid)
	F	73	Severe (hospitalized)	2–3 weeks	Tremors, impaired posture and balance with repeated falls, loss of spontaneous movements, moderate cogwheel rigidity, hypomimia	Not reported	None	Modest response to levodopa/carbidopa, no significant motor function improvement

Pt, Patient; M, male; F, female; ICU, Intensive care unit.

In addition to COVID-19-associated acute parkinsonism, COVID-19 survivors developed post-COVID chronic parkinsonism with an estimated incidence of 0.11% (1), and Table 4 provides a summary of the incidence and case number details. PD motor symptoms such as tremors (10.8%) and rigidity (5.38%) were reported in COVID-19 patients with motor symptoms (77). In long COVID, a meta-analysis found that 44% of COVID-19 survivors experienced functional mobility impairment 5 months after the disease onset (13). Moreover, sensorimotor symptoms (including tremors and internal vibrations) were prevalent among COVID-19 patients 7 months after the onset of the sickness, and there was an increasing trend of tremors 6–7 months after COVID-19 (34).

**Table 4:** Summary of the estimated incidences and numbers of post-COVID-19 parkinsonism reported in Taquet et al. (1).

	**Number of COVID-19 survivors**	**Estimated incidences of parkinsonism**	**Number of post-COVID-19 parkinsonism cases**	**Percentage of case numbers in each category over total case number**
For all in the cohort	236,379	0.11%	260	100%
For those who were hospitalized	46,302	0.20%	93	35.8%
For those who had ICU care	8,945	0.26%	23	8.8%
For those who had encephalopathy	6,229	0.46%	29	11.2%

ICU, Intensive care unit.

Further, smell impairment (olfactory dysfunction) is an early non-motor symptom of PD, while taste impairment can appear in early PD but more frequently in advanced PD (78). In addition, cognitive impairment (including memory problems), gastrointestinal problems and sleep disorders are common non-motor symptoms of PD. Since SARS-CoV-2 infection induces neuroinflammation and PD neurodegeneration and increases individual vulnerability to PD or parkinsonism (1, 7, 26, 47), the high prevalence of olfactory dysfunction and taste disorder in COVID-19 patients and the frequently reported cognitive impairment, functional mobility impairment, tremors, internal vibrations, gastrointestinal problems and sleep disorder in long COVID (13, 34, 35) suggest possible future development of PD or parkinsonism in patients with long COVID.

The relatively low incidence of parkinsonism in COVID-19 patients or survivors (1) may be because the nigrostriatal damage caused by SARS-CoV-2 infection (77) in some patients (especially those with long COVID) or COVID-19 survivors was not severe enough to manifest PD or parkinsonism symptoms (i.e., they were at the prodromal or presymptomatic stage of PD or parkinsonism) and thus they remained undiagnosed/unreported (13, 28, 35). Therefore, long-term following up on COVID-19 patients who have non-motor and/or motor symptoms of PD (or parkinsonism) will help reveal the true incidence of PD (or parkinsonism) in patients with long COVID (1, 27, 28, 77).

## Mechanisms of neurological injury in the infectious diseases and neurodegenerative disorders and treatments of these diseases

### Mechanisms of neurological injuries in the diseases

The mechanisms of neurological injury caused by infectious diseases (COVID-19, influenza, and prion disease), and the neurodegenerative disorders are summarized in Table 5. For COVID-19 and PD, the mechanisms underlying their neurological symptoms are highlighted in this section.

**Table 5:** Mechanisms and treatments of neorlogical injury in the infectious diseases (COVID-19, influenza and prion disease), and the neurodegenerative disorders (Alzheimer's Disease, Parkinson's Disease, and Amyotrophic Lateral Sclerosis).

	**COVID-19 disease**	**Influenza**	**Prion disease**	**AD**	**PD**	**ALS**
Mechanism	Not fully understood; 1) Viral neuroinvasion; (1) Direct invasion through peripheral nerves (e.g., olfactory nerve), or the hematogenous route (e.g., vascular endothelium); (2) Indirect invasion through systemic inflammation, cytokine storm, coagulation or autoimmune response which caused neuroinflammation, oxidative stress, hypoxia, and nerve cell apoptosis (79, 80) 2) Host neuroimmune responses; 3) Viral-infection-induced coagulation, cerebrovascular diseases and bioenergy failure (79, 81). Long COVID may be related to SARS-CoV-2-induced autoimmunity (82), prolonged inflammation (viral fragments), mitochondrial dysfunction, oxidative stress and gut microbiota alteration (83).	Unclear; (1) Direct neuronal damage through peripheral nerves or the hematogenous route; (2) Indirect neuronal damage through systemic inflammation, cytokine storm, coagulation or autoimmune response which caused neuroinflammation, oxidative stress, hypoxia, and nerve cell apoptosis (79, 84)	Unclear; Prions enter the gut lumen and the enteric nervous system through nerve fiber endings, then reach the CNS through autonomic nerves. Prions replicate in the CNS and form prion aggregation (amyloid plagues) which activates microglia and causes neuroinflammation that initiates neurodegeneration (85). In addition, prions may cause a dysbiosis in the gut which produces microbial amyloid that activates the immune system to enhance microglia activation and neuronal amyloid production and accumulation in the brain (86). Further, a pathological mutation (T183A) significantly enhanced prion misfolding and aggregation (87).	Not fully understood; Key mechanisms include beta-amyloid and tau misfolding and aggregation, mitochondrial dysfunction, oxidative stress, impairment of protein clearance, and neuroinflammation. Infections with pathogens (e.g., HSV-1 or oral porphyromonas gingivalis or gut microbiota) and exposures to metal ions can trigger beta-amyloid aggregation (3, 88, 89), which further induces tau aggregation (90) and neuroinflammation, leading to neuronal degeneration and loss in the hippocampus and associated regions (91)	Unclear; Key mechanisms include alpha-synuclein misfolding and aggregation, mitochondrial dysfunction, oxidative stress, impairment of protein clearance, and neuroinflammation. Abnormal dopamine metabolism, infections with pathogens (e.g., influenza A viruses or gut microbiota), and exposures to pesticides, heavy metals and toxic chemicals can induce abnormal alpha-synuclein aggregation (92), which induces microglia activation and neuroinflammation, and triggers an autoimmune response that induces immune cells to attack dopaminergic neurons in the substantia nigra (93), leading to dopamine neuron degeneration (or loss) and dopamine deficit (or depletion) in the brain (94)	Unclear; Key mechanisms include TDP-43 misfolding and aggregation, mitochondrial dysfunction, oxidative stress, impairment of protein clearance, defective RNA processing, glutamate excitotoxicity, glial dysfunction, nucleocytoplasmic transport defects, axonal transport disruption, impaired DNA repair, and neuroinflammation. Infections with pathogens (e.g., EV or HIV) can trigger TDP-43 upregulation or aggregation (95, 96). These cause progressive degeneration and loss of motor neurons (97).
Treatment	No cure yet; Antiviral drugs such as Paxlovid (nirmatrelvir + ritonavir), Veklury (Remdesivir) and Molnupiravir; Monoclonal antibodies such as Bamlanivimab; Supportive care (fluid therapy, oxygen support, etc.); Vaccine can prevent COVID-19	No cure yet; Post-exposure prophylaxis with the antiviral drugs (oseltamivir, zanamivir, etc.); Anti-inflammatory therapy; Supportive care; Vaccine (flu shot) can prevent influenza	No cure yet; Supportive care (e.g., use drugs to relieve pain, ease muscle spasms, or to reduce psychiatric symptoms)	No cure yet; Aducanumab (to reduce beta-amyloid plaques); Cholinesterase inhibitors (Donepezil or Aricept, Rivastigmine or Exelon, and Galantamine or Razadyne to improve memory and thinking); Glutamate regulators (Memantine or Namenda to improve memory, attention, language, etc.); Cholinesterase inhibitor + glutamate regulator (Donepezil and memantine, or Namzaric); Orexin receptor antagonist (Suvorexant or Belsomra to treat insomnia)	No cure yet; Levodopa and dopamine agonists (such as Ropinrole and Pramipexole) (to reduce motor symptoms); MAO-B inhibitors (e.g., Safinamide or Xadago to alleviate motor symptoms); Antidepresssants (to treat PD-associated depression); Deep brain stimulation (to improve motor function and reduce tremors); Stem cell therapy (to repair or replace damaged dopaminergic neurons)	No cure yet; Riluzole (Rilutek to reduce glutamate level to reduce damage to motor neurons); Edaravone (Radicava, to slow function decline); Gabapentin pregabalin, acetaminophen (to reduce pain); Physical and occupational therapy; Communication, nutrition and breathing support

AD, Alzheimer's disease; ALS, Amyotrophic Lateral Sclerosis; BBB, blood-brain barrier; CNS, central nervous system; EV, Enterovirus; HSV-1, Herpes simplex virus type 1; MAO-B, Monoamine oxidase B; PD, Parkinson's disease.

In general, there are several ways that pathogens in infectious diseases can cause neuronal damage which leads to nervous system impairment and neurological symptoms. (1) Direct invasion which can lead to neuroinflammation, encephalitis, meningitis and myelitis; (2) Indirect invasion or para-infectious complications such as sepsis, metabolic dysfunction and coagulopathy, which can lead to encephalopathy, seizures, vasculitis and stroke; (3) Infection-triggered immune-mediated attacks in the nervous system which can lead to acute disseminated encephalomyelitis (ADEM), acute motor axonal neuropathy, acute inflammatory demyelinating polyneuropathy, and Guillain–Barré syndromes (GBS); (4) Persistent or latent infection (due to, e.g., viral mutation) and/or interplay with other pathogens, which can lead to potential late reactivation, neurodegeneration, neurocognitive disorders and subacute sclerosing encephalitis (79).

In COVID-19, the mechanisms of the neurological symptoms have not been fully established and more research is needed to unfold them. Potential mechanisms include viral neuroinvasion, host neuroimmune responses, viral-infection-induced coagulopathy, cerebrovascular diseases (such as microvascular injury, vasculitis, and ischemic or hemorrhagic stroke), and bioenergy failure (30, 79–81). There are mainly 2 ways of SARS-CoV-2 neuroinvasion that causes damage to the human nervous system: (1) Direct neuronal damage that SARS-CoV-2 virus may travel to the brain through peripheral nerves such as the olfactory nerve and the vagus nerve, or through the hematogenous route to cross the blood-brain-barrier (BBB) (80, 98). (2) Indirect neuronal damage through systemic inflammation or cytokine storm or autoimmune response triggered by a viral infection, which caused metabolic dysfunction, oxidative stress, hypoxia, neuroinflammation and neuronal death (79, 80, 99, 100). As to long COVID, potential mechanisms may include SARS-CoV-2-induced autoimmunity (82), prolonged inflammation (due to unresolved viral fragments and delayed viral clearance), mitochondrial dysfunction, oxidative stress and gut microbiota alteration (83). In addition, it has been found that factors such as SARS-CoV-2 RNA level in the blood, the presence of autoantibodies and reactivation of the Epstein-Barr virus (EBV) increase the risk for long COVID (101).

The mechanisms of PD have not been fully understood, but key molecular mechanisms include alpha-synuclein misfolding and aggregation, mitochondrial dysfunction, oxidative stress, impairment of protein clearance, and neuroinflammation (94). Excessive oxidation of dopamine can induce mitochondrial dysfunction and oxidative stress, which leads to misfolded alpha-synuclein aggregation (102). In addition, infections with pathogens (e.g., influenza A viruses or gut bacteria), and exposures to pesticides, heavy metals, and chemicals such as 1-methy1-4-phenyl-1,2,3,6-tetrahydropyridine (MPTP) can also cause abnormal alpha-synuclein aggregation (5, 92). which activates microglia and may trigger an autoimmune response that induces immune cells to attack dopaminergic neurons in the substantia nigra (93). Alpha-synuclein pathology and microglial activation, along with other abnormalities such as impairment of protein degradation and clearance, mitochondrial dysfunction, oxidative stress and glutaminergic excitotoxicity, lead to neuroinflammation in the substantia nigra. Neuroinflammation initiates neurodegeneration, which further causes neuronal dysfunction and death of dopamine neurons in the substantia nigra, resulting in dopamine deficit (or depletion) and motor symptoms in PD patients (94). Further, alpha-synuclein aggregates in the olfactory system and the enteric nervous system propagate to the brain through the olfactory nerve and the vagus nerve in a prion-like manner, while short-chain fatty acids and bacteria metabolites (produced by gut microbiota) may reach the brain through the endocrine route to induce alpha-synuclein aggregation and microglia activation in the brain (92, 103). Based on pathological findings, the Braak hypothesis (104, 105) and prion-like mechanisms in PD have been proposed (106), and they are discussed in the Discussion section.

**Figure 1:**
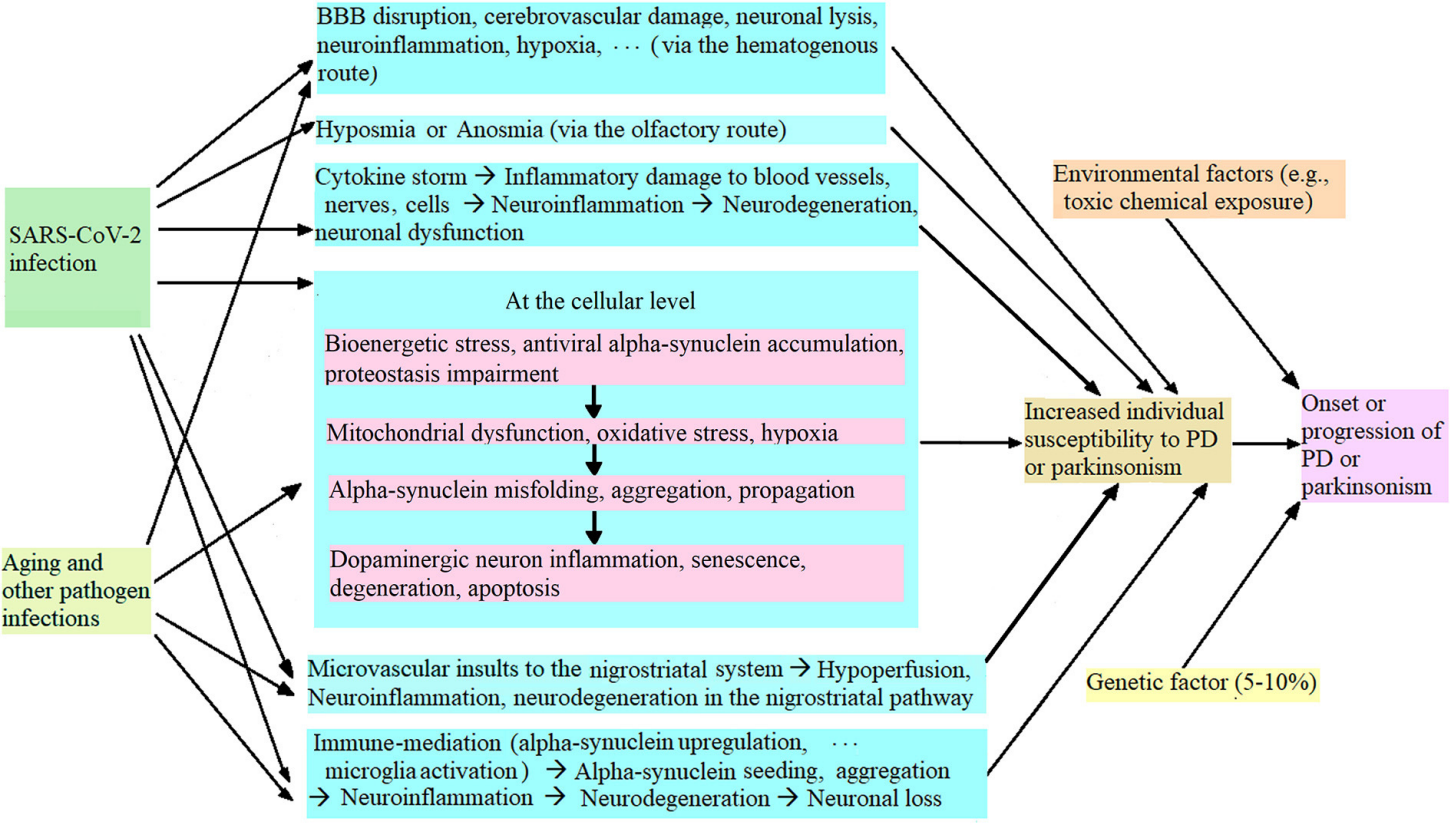
Potential mechanism of SARS-CoV-2 infection leading to increased individual vulnerability to PD or parkinsonism.

During the COVID-19 pandemic, it has been found that SARS-CoV-2 infection increases individual susceptibility to PD or parkinsonism (9, 24), and the potential mechanism is illustrated in Figure 1. Briefly, SARS-CoV-2 infection can cause blood-brain-barrier (BBB) disruption, cerebrovascular damage, neuronal lysis, neuroinflammation, etc. (*via* the hematogenous route), and hyposmia or anosmia (*via* the olfactory route) (80, 98). SARS-CoV-2 infection triggers a cytokine storm that induces oxidative stress and neuroinflammation, which further initiated neurodegeneration (80, 99, 100). At the cellular level, SARS-CoV-2 infection causes bioenergetic stress, alpha-synuclein upregulation, antiviral alpha-synuclein accumulation, and proteostasis impairment in the infected dopaminergic neurons in the substantia nigra, which induces mitochondrial dysfunction, oxidative stress and hypoxia (24, 107–111). In addition, SARS-CoV-2 infection triggers microvascular injury (such as perivascular activated microglia, astrocytosis, and CD8+ T cell infiltration) in the basal ganglia and brain stem (47, 49) and (activated microglia and neuronophagia) in the substantia nigra (47) which results in hypoperfusion, neuroinflammation and subsequent neurodegeneration. These damages to the nervous system induce alpha-synuclein (or Lewy-body-like) pathology (110, 112), trigger neuroinflammation and increase the dopaminergic neurons' vulnerability to neuroinflammation, which leads to dopaminergic neuron senescence and neurodegeneration (9, 24, 108). SARS-CoV-2 nucleocapsid protein accelerated alpha-synuclein aggregation, which induced neuroinflammation and subsequent neurodegeneration, and increased cell death (9, 23). At the neuroimmune level, SARS-CoV-2 infection can activate microglia by causing alpha-synuclein pathology, trigger neuroinflammation through immune mediation, and induce neurodegeneration and dopaminergic neuron loss in the substantia nigra, which increases individual susceptibility to PD or parkinsonism, leading to acute or chronic parkinsonism (1, 9, 26, 31). Further, aging, infections with other pathogens (e.g., flu virus), genetic factors and/or environmental factors (such as toxic chemicals and metals) can synergize to worsen nigral neurodegeneration and dopaminergic neuronal loss, and induce or exacerbate PD or parkinsonism (25, 32, 113).

Bioinformatics analysis has confirmed that SARS-CoV-2 (spike and nucleocapsid) proteins have a favorable binding affinity to alpha-synuclein, and a cell line study has verified that SARS-CoV-2 proteins upregulate alpha-synuclein expression and induce alpha-synuclein aggregation and Lewy-body-like pathology (110). Therefore, SARS-CoV-2 infection could change normal alpha-synuclein proteins and turn them into pathological (misfolded, aggregated) alpha-synuclein that seeding and spreading like prions. This is further discussed in the Discussion section.

### Treatments of the diseases

Currently, there is no cure for the six diseases covered in this paper (infectious diseases: COVID-19, influenza and prion diseases; and neurodegenerative disorders: AD, PD and ALS), but vaccines for COVID-19 and influenza are available. Table 5 outlines the treatments and prevention methods of these diseases. Treatments for these incurable diseases are mainly medications (to manage symptoms or slow down disease progression) and supportive care. For example, in COVID-19, new antiviral drugs (such as Paxlovid), monoclonal antibodies (such as Bamlanivimab), and supportive care have been used to treat COVID-19 symptoms, relieve patients from suffering, and save their lives; while in PD, medications such as levodopa can reduce motor symptoms, and surgical procedures (e.g., deep brain stimulation) are effective in reducing tremors and improving motor function. Further, new therapies such as stem cell therapy are promising in repairing or replacing damaged dopaminergic neurons in PD (114).

## Discussion

Infectious diseases such as COVID-19 and influenza are very different from neurodegenerative disorders such as AD, PD and ALS, but some common neurological symptoms (e.g., viral infection with SARS-CoV-2 or influenza A viruses can induce symptoms of AD, PD, or ALS) bring the two groups of diseases together. Examining the common neurological symptoms and their mechanisms reveals interconnections between the two groups of diseases.

### The relationship between infectious diseases and neurodegenerative disorders

Since pathological proteins such as beta-amyloid, tau, alpha-synuclein and transactive response DNA binding protein 43 (TDP-43) in AD, PD or ALS act like prions in misfolding, aggregating, seeding, and spreading in the brain, these neurodegenerative disorders have been called prion-like diseases (115–117). In addition, prion diseases (such as Kuru and Creutzfeldt-Jakob disease) are both infectious diseases and neurodegenerative disorders. Thus, prion diseases and prions are the links between infectious diseases and neurodegenerative disorders.

Traditionally, pathological hallmarks of AD, PD, and ALS such as senile plaques (beta-amyloid aggregates), neurofibrillary tangles (tau aggregates), Lewy bodies (alpha-synuclein aggregates), and Bunina bodies (TDP-43 aggregates) were considered as non-transmittable. Recent research findings have changed the traditional opinion (115, 118–121). The transmissibility of pathological proteins such as alpha-synuclein, beta-amyloid, tau and TDP-43 has been reported, and these proteins can be transmitted between cells in the central nervous system (CNS), propagate across species (e.g., transmitted PD-associated α-synuclein seeds could propagate in new mammal host) (119), and even spread between individuals (e.g., AD-associated pathological beta-amyloid) (122), like prions in prion diseases (122–126).

Further, recent findings indicated that proteins such as beta-amyloid and alpha-synuclein are antimicrobial peptides protecting neurons against microbes (bacteria, fungi, viruses, etc.) (127), restricting viral infections in the brain (128), and acting like chemoattractants to immune cells (e.g., monocytes) (129). In addition, hyperphosphorylation of certain residue of tau can protect against beta-amyloid-induced excitotoxicity (e.g., due to antiviral beta-amyloid accumulation) and spine loss (130), while TDP-43 has a positive role in antiviral response (131) and infection with enterovirus (EV) or HIV can induce TDP-43 upregulation or aggregation (96, 132). These findings suggest that pathogen infection can trigger the upregulation, accumulation, misfolding and aggregation of the antimicrobial proteins such as beta-amyloid, alpha-synuclein and TDP-43, which consequently activates microglia in the brain. Namely, pathogen infections can induce the accumulation of the antimicrobial proteins and initiate the formation of “prions” that turns these normal proteins into pathological prion-like proteins, which further evokes neuroinflammation and subsequent neurodegeneration in the neurodegenerative disorders.

Compelling evidence has shown that in AD, infections with pathogens such as herpes simplex virus 1, oral porphyromonas gingivalis, and abnormal gut microbiota can induce anti-pathogen accumulation of beta-amyloid, cause beta-amyloid to misfold and aggregate which triggers tau aggregation (90), and thus these pathogen infections evoke the formation of “prions” that conforms normal beta-amyloid and tau proteins to pathological prion-like beta-amyloid and tau proteins (3, 88, 89). In PD, infections with pathogens such as influenza A virus, SARS-CoV-2, West Nile virus, and gut bacteria (e.g., Desulfovibrio bacteria) can trigger an anti-pathogen accumulation of alpha-synuclein and alpha-synuclein pathology (misfolding, aggregation, etc.), and evoke “prion” formation that conforms normal alpha-synuclein proteins to pathological prion-like alpha-synuclein proteins (5, 23, 92, 109, 111, 133, 134). In ALS, infections with pathogens such as EV and HIV can induce TDP-43 upregulation, misfolding and aggregation, and turn normal TDP-43 into pathological prion-like TDP-43 proteins (6, 95, 96, 132) and infection with gut microbiota could modulate ALS (135). Further, in prion diseases, infection with prions (PrP^Sc^) may cause a gut dysbiosis which produces microbial amyloid that activates the immune system, triggers misfolding and aggregation of prion protein PrP^C^ in the brain, conforms normal PrP^C^ to pathological prions (PrP^Sc^), activates microglia, and enhances neuronal amyloid production and accumulation (136).

Interestingly, gut microbiota abnormality and gut-brain axis alterations have been implicated in the pathogenesis of neurodegenerative disorders such as AD, PD, ALS, and prion diseases (88, 92, 135, 136). In addition, a pathogen-infection-evoked autoimmune response has been found in AD, PD, ALS, and prion diseases that cause the immune system to treat pathological prions (or “prions”) as pathogens and attack neurons that have the presence or aggregation of these pathogens (137–140). Therefore, the pathogenesis of neurodegenerative disorders may be a 2-step relay process: First, one or multiple pathogen infections trigger an anti-pathogen accumulation of antimicrobial proteins (beta-amyloid, alpha-synuclein, TDP-43, etc.) which evokes the formation of pathological prions (or prion-like proteins), and induces neuroinflammation; Second, the pathological prions or prion-like proteins keep seeding, spreading and conforming (normal proteins to prions or prion-like proteins), which triggers persistent neuroinflammation and subsequent neurodegeneration in these neurodegenerative disorders.

### The unknown pathogen in the Braak hypothesis in sporadic PD

Braak et al. have hypothesized that sporadic PD is caused by an unknown pathogen that enters the body *via* the respiratory route, is swallowed subsequently, and reaches the gut, initiating Lewy pathology in the olfactory and the digestive tracts (104). Further, Braak staging has been developed for AD (141, 142) and PD (105).

Over the years, mounting evidence from clinical research has supported the Braak hypothesis (143). In AD, it has been found that tau-PET and Braak staging for AD are prognostic markers of cognitive decline in patients with mild cognitive impairment or dementia including AD (144). In PD, Lewy bodies have been found in the neurons of the olfactory tract and the enteric nervous system in patients with PD (which leads to olfactory/smell impairment and digestive/gastrointestinal disorder at the early stage of PD), and the presence of Lewy bodies in the olfactory tract and the enteric nervous system precedes the onset of PD motor symptoms (145, 146).

The Braak hypothesis has also been supported by animal studies with influenza viruses (5, 113). Jang et al. showed that (1) the influenza A virus H5N1 traveled to the brain (of mice) through the vagus nerve, induced alpha-synuclein phosphorylation and aggregation, and persistent microglial activation (neuroinflammation), which triggered neurodegeneration, dopaminergic neuronal loss in the substantia nigra and parkinsonism, and (2) the spread of the influenza virus in the brain was similar to the alpha-synuclein progression pattern described in the Braak staging in sporadic PD (5). Influenza A virus H1N1 also infected dopaminergic neurons and its replication in neurons can induce seeding of aggregated alpha-synuclein, leading to alpha-synuclein aggregates and synucleinopathies (147). In addition, influenza A virus H1N1 activated the immune system that further triggered neuroinflammation, and thus infection with influenza A virus H1N1 made dopaminergic neurons susceptible to toxic chemicals such as MPTP (113). Therefore, multiple exposures to influenza A viruses (H1N1, H5N1, etc.), other pathogens (SARS-CoV-2, gut microbes, etc.) and toxic chemicals over years or decades can synergize to induce PD (113).

The unknown pathogen that causes sporadic PD in the Braak hypothesis is not one pathogen, but a relay of pathogens such as microbes (influenza A viruses, SARS-CoV-2, Desulfovibrio bacteria in gut microbiota, etc.) and prion-like alpha-synuclein proteins. Like a relay, infections with the microbes initiate the formation of alpha-synuclein “prions” which keep seeding, spreading and conforming (normal alpha-synuclein proteins to pathological prion-like alpha-synuclein proteins), inducing neuroinflammation and subsequent neurodegeneration, and causing damage and neuronal loss to dopaminergic neurons in the substantia nigra in sporadic PD.

### Therapeutic and preventive implications in the incurable neurodegenerative disorders

Since infections with a relay of pathogens (microbes and prions or prion-like proteins) over time (months, years or decades) can synergize to induce devastating neurodegenerative disorders such as sporadic PD and AD, a new direction has emerged for the treatment and prevention of these neurodegenerative diseases, i.e., treating or preventing these diseases by treating or preventing the infections caused by the pathogens. This insight has brought new light and hopes to the field, which has suggested that neurodegenerative disorders may be curable using a systematic approach that treatments targeting a relay of pathogens including microbes and prions or prion-like proteins need to work together in a systematic, orderly and harmonious way in order to provide effective cures for these disorders. Thus, the treatment or prevention of these diseases may be two-fold.

First, treat or prevent the infections and replications of the microbes in the human body to remove the source of “prion” production in these neurodegenerative disorders.

In AD, a recent study showed that antiviral treatment (such as acyclovir and valaciclovir) for infection of herpes viruses such as HSV-1 was associated with a lower long-term risk of dementia (including AD), while untreated herpes infection was related to a higher risk (148). A clinical trial is also investigating whether the antiviral drug valaciclovir can slow the progression of AD in patients with HSV-1 infection (149). In addition, recent research has found that oral porphyromonas gingivalis infection secreted toxic protease gingipains and increased beta-amyloid production, and gingipains inhibitor reduced the infection, beta-amyloid aggregation and neuroinflammation (89). Further, vaccines against these microbes (e.g., HSV-1) are promising to prevent AD induced by such microbe infections.

In PD, it has been reported that treating or preventing infections caused by microbes such as influenza A viruses, SARS-CoV-2, and gut microbiota can protect dopaminergic neurons or reduce the risk of PD induced by such infections (86, 113, 150). Research showed that influenza vaccine (flu shot) or anti-viral medication treatment could protect against dopaminergic neuronal loss in the substantia nigra (caused by influenza A virus infection or MPTP toxicity) (113). Similarly, since COVID-19 vaccines protect against SARS-CoV-2 infection, they may help prevent COVID-19-associated parkinsonism by preventing COVID-19 or SARS-CoV-2 infection. Further, treatment for COVID-19 varied in patients with COVID-19-associated acute parkinsonism: one patient had spontaneously improved motor symptoms after treatment with convalescent plasma for SARS-CoV-2 infection (19), while another patient (who did not respond to dopaminergic medication) achieved significant spontaneous improvement in motor symptoms without any specific treatment, suggesting that treatment for SARS-CoV-2 infection in this case came from the immune system of the patient (16).

In addition, research has shown that since the introduction of highly active antiretroviral therapy (HAART), the overall prevalence of neurological symptoms including HIV-associated dementia dropped by over 60% (151, 152). Antiretroviral therapy also reversed HIV-induced parkinsonism (153) and HIV-induced ALS (6). Similarly, antiviral drug ribavirin could reverse EV-infection-induced ALS symptoms in mice (132).

Second, treat or prevent propagation of prions or prion-like proteins by reducing or removing prions or prion-like proteins and mediators of neuroinflammation and neurodegeneration in the neurodegenerative disorders, and providing neuroprotection and immuno-modulation to prevent or reverse the neurodegeneration process and restore normal protein homeostasis.

Immunotherapy has been introduced to neurodegenerative disorders in recent years. It uses either passive immunotherapy monoclonal antibodies, or active immunotherapy therapeutic vaccines (that induce adaptive immune responses to produce antibodies) to reduce or remove prions or prion-like proteins and/or mediators of neuroinflammation and neurodegeneration in the neurodegenerative disorders in order to manage or prevent the diseases.

In AD, a beta-amyloid immunotherapy Aducanumab or Aduhelm (an anti-beta-amyloid human antibody) that can reduce beta-amyloid plaques has been approved by the FDA, and other similar therapeutics such as gantenerumab are in clinical trials (154). In addition, therapeutic beta-amyloid vaccines have been developed to train the immune system to produce beta-amyloid antibodies, and beta-amyloid vaccines (such as ABvac 40 by Araclon Biotech) have entered clinical trials (154). As to tau, clinical trials of tau immunotherapies that use tau-targeted antibodies (such as semorinemab) to reduce phosphorylated tau levels in AD have started, and tau vaccines such as ACI-35 (by AC Immune) that stimulate the body to develop tau antibodies have also entered clinical trials (154, 155). Currently, the clinical efficacy of beta-amyloid or tau immunotherapies in restoring cognition (e.g., memory improvement) in AD patients is limited. Rather than target pathological beta-amyloid or tau alone, another strategy is to address the pathological imbalance in pathways that created the abnormal beta-amyloid and tau aggregation and modulate the immune system to reverse the AD neurodegeneration process and restore normal protein homeostasis. AD immune system-modulating vaccines or medications such as GV1001 (by GemVax and KAEL Bio) can stimulate the immune system to clear beta-amyloid plaques and tau tangles, protect neurons, and improve cognition in AD, which are being studied in clinical trials (154).

In PD, ENT-01 (by Enterin Inc.) reduces alpha-synuclein accumulation in the neurons in the gut, restores enteric neuron functions, and repairs the dysfunctional gut-brain axis in PD, and a clinical trial was completed in 2018 which treated constipation in over 80% patients, and revealed the reversal of a neurodegenerative process in PD patients for their symptoms (156). In addition, alpha-synuclein immunotherapy using alpha-synuclein antibodies (such as BIIB054) has entered clinical trials, and alpha-synuclein vaccines (that prompt the body to produce alpha-synuclein antibodies) such as PD01A (by AFFiRiS), ACI-7104 (by AC Immune), and UB312 (by Vaxxinity) are being studied in clinical trials (157, 158).

In ALS, a recent study found that thioridazine enhanced the clearance of TDP-43 aggregates which recovered TDP-43 functionality (159). In addition, targeting inflammatory or neurodegenerative mediators, immunotherapy using drugs such as granulocyte-macrophage colony-stimulating factor (GM-CSF) is promising to reduce TDP-43-induced neuroinflammation and motor neuron death, which may slow ALS progression and enhance patient survival (160).

## Conclusion

In summary, the neurological symptoms of the infectious diseases (COVID-19, influenza and prion disease) and the neurodegenerative disorders (AD, PD, and ALS), the potential mechanisms underlying these symptoms, and the relationship between the two groups of diseases shed light on the treatment and prevention of the currently incurable neurodegenerative disorders. For example, SARS-CoV-2 infection can increase individual vulnerability to PD or parkinsonism, and infections with a relay of microbes (influenza A viruses, SARS-CoV-2, gut microbiota, etc.) and prion-like alpha-synuclein proteins over time can synergize to induce PD or parkinsonism. Therefore, a systematic approach that targets these pathogens and the pathogen-induced neuroinflammation and neurodegeneration may provide cures for the neurodegenerative disorders like PD. This approach includes: (1) treating or preventing the infections and replications of the microbes, and (2) treating or preventing propagation of prions or prion-like proteins as well as the neuroinflammation and subsequent neurodegeneration induced by the pathology of prions or prion-like proteins, and restoring normal protein homeostasis. Further, antiviral/antimicrobial medications, vaccines, immunotherapies and new therapies (such as stem cell therapy) need to work together to treat or prevent devastating neurodegenerative disorders. Effective immunotherapies for AD, PD, and ALS (including monoclonal antibodies and therapeutic vaccines for prion-like proteins such as beta-amyloid, tau, alpha-synuclein and TDP-43) are on the horizon. As medical science and technology advance rapidly, better COVID-19 vaccines for SARS-CoV-2 variants, effective antiviral or antimicrobial drugs, innovative immunotherapies (such as monoclonal antibodies for prion-like alpha-synuclein proteins and therapeutic vaccines for PD or parkinsonism), as well as new (or unconventional) therapies will be developed and made available in the near future, which will help prevent a possible epidemic or global spreading of post-COVID-19 parkinsonism in the 21st century.

## Author contributions

JZ contributed to the research design, data collection and analysis, and drafting of the manuscript.
